# Changes in Work-Family Conflict of Chinese Employees: A Cross-Temporal Meta-Analysis, 2005–2016

**DOI:** 10.3389/fpsyg.2020.00124

**Published:** 2020-02-14

**Authors:** Sufei Xin, Yang Zheng, Ziqiang Xin

**Affiliations:** ^1^School of Education Science, Ludong University, Yantai, China; ^2^School of Sociology and Psychology, Central University of Finance and Economics, Beijing, China

**Keywords:** Chinese employees, work-family conflict, cross-temporal meta-analysis, stress, social changes

## Abstract

With the rapid growth of China's economy, work-family conflict (WFC) level of Chinese employees might have changed over time. The present research performed a cross-temporal meta-analysis of 71 papers using the Work-Family Conflict Scale (WFCS) from three Chinese academic databases and three databases in English to investigate changes in Chinese employees' WFC (*N* = 23,635) during 2005–2016. Results showed that the WFC level of employees increased significantly by 0.77 standard deviations over the past 12 years. The increasing trend over time occurred among both male and female employees, which is slightly more salient among male employees. However, there was no significant gender difference in WFC scores. This study found that the increase in WFC scores among Chinese employees was associated with scores of six social indicators that might cause stress in workplace (the number of employees and number of college graduates) and stress in family (divorce rate, residents' consumption level, elderly dependency ratio, and family size) of 5 years before and the year of data collection, which indicates that social changes played an important role in changes of WFC. The explanations and implications of these changes are discussed.

## Introduction

People in modern societies are faced with difficulties in balancing weight of their work and family, which has been considered not only as a broader social phenomenon but also as a notable psychological one (Sǎucan et al., 2015). Since Greenhaus and Beutell (1985) first defined Work-Family Conflict (WFC) as a failure in building compatible relationship between work and family roles, numerous studies have been conducted with this remarkable social concern (e.g., Duxbury et al., 1994; Aycan and Eskin, 2005). It was found that the increase in the sense of WFC among American employed parents between 1977 and 1997 (Nomaguchi and Johnson, 2009). In China, the traditional Chinese large family system could be collapsed by the industrialization processes (Tian, 2002), leading to a general increase in the number of dual-earner couples (Tong and Liu, 2015). Consequently it might be more difficult for Chinese employees to balance time allocation between work and family. Moreover, China has experienced a surprisingly growing annual GDP with an average rate of 10% (Xin and Xin, 2016), in which employees play a crucial role. The number of employees in China is also increasing continuously by ~13.97% from 1997 to 2016 (National Bureau of Statistics of China., 2017). Therefore, it is necessary to examine the influence of these critical social changes in WFC perceived by Chinese employees in China.

## Changes in Chinese Employees' Work-Family Conflict Over Time

WFC is a conflict between roles that occurs when the demands of work or family make it difficult to fulfill the demands in the other roles (e.g., Carlson et al., 2000; Promislo et al., 2011). Conflict can occur in two directions (e.g., French et al., 2018): family interfering work (FIW) and work interfering family (WIF). In recent years, many studies on WFC and its influence have been published in academic journals. Previous research showed that WFC has crucial effects on physical-mental health and work-family satisfaction (Eby et al., 2005; Zhang, 2009). It has also been confirmed that WFC is associated with higher turnover intention and absenteeism rates (Kossek and Ozeki, 1998), which makes it difficult for individuals to take part in family (Duxbury and Higgins, 1991). Since the 1980s, the rapid growth of China's economy might make it more difficult to balance time allocation between work and family for employees. However, no study examines how WFC among Chinese employees has varied with birth cohort. Therefore, it is important to investigate the changes in WFC of Chinese employees over time, which will also provide important reference for other countries with rapid economic development worldwide.

Along with social changes, WFC of employees might have changed across birth cohort. Previous studies have found that birth cohort is an important proxy for macro-level social environment of different time periods that might have substantial effects on individuals' psychological traits (e.g., Stewart and Healy, 1989; Twenge, 2000). Studies by Twenge and her colleagues showed strong birth cohort differences in Americans' anxiety (Twenge, 2000), self-esteem (Twenge and Campbell, 2001), and narcissism (Twenge and Foster, 2010). In China, Xin and his associates have found that psychological functioning (i.e., anxiety, mental health, and loneliness) of adolescents changed over time (Xin and Zhang, 2009; Xin et al., 2010; Xin and Xin, 2016). All the above studies indicated the important role social changes might play in individuals' psychological status. WFC, as an important negative indicator relating to individuals' psychological well-being (e.g., Hong et al., 2013), has never been studied with regards to its cross-temporal changes. Considering the mean score of WFC was 2.52 in 2005 (Gan, 2007) and 4.08 in 2015 reported in a recent cross-sectional study by Zhang and Sun (2017), it may be reasonable to argue that the WFC level of Chinese employees have increased since the early 2000s.

WFC is generally assessed using classic self-report inventories such as the Work-Family Conflict Scale (WFCS) (Carlson et al., 2000), which is well-developed and found with sound psychometric properties in prior research (Promislo et al., 2011). The Chinese version of the WFCS is the most popular scale for measuring WFC of Chinese employees, with high validity and reliability (Cronbach's α coefficients ranging from 0.84 to 0.90, e.g., Bian et al., 2017; Ding et al., 2017; Hao et al., 2017). A large body of previous studies adopting the scale that measures WFC among Chinese employees across different birth cohorts has been published in different years (e.g., Gan, 2007; Chen et al., 2010; Li, 2012; Hao et al., 2017), which makes it rather convenient to investigate how WFC scores on the scale have changed over time by using a cross-temporal meta-analysis.

We conducted a cross-temporal meta-analysis for the present study to examine changes in WFC of Chinese employees. Instead of computing an effect size for each study, cross-temporal meta-analysis locates samples of the similar age who completed the same psychological scales at different time points, and then analyzes the relationship between mean scores on the scale (e.g., WFCS) and the year of data collection (e.g., Twenge, 2000; Twenge and Im, 2007; Xin et al., 2010; Xin and Xin, 2016).

## The Association Between Work-Family Conflict and Social Changes Over Time

In the past few decades, dramatic macro social changes have occurred in China due to the rapid development of economy. The cross-temporal meta-analysis assumes that social statistical indicators can be used to reflect macro social changes and partially explain individual psychological changes (e.g., Twenge, 2000; Xin and Xin, 2016). Previous research found that WFC level of employees is mainly affected by dual-stress from work and family (French et al., 2018). On the one hand, regarding *stress from work*, the job strain model (e.g., Karasek, 1979) has been one of the most influential theories in research on work characteristics and WFC. Evidence continues to accumulate that high job strain is associated with high level of WFC (e.g., Wang, 2005; Zhao, 2011). Chinese employees experienced increasing stress from employment competition in recent decades: the number of employees and college graduates has increased ~2- and 48-fold, respectively, since 1980s (National Bureau of Statistics of China., 2017). In order to cope with such stress, Chinese employees might sacrifice family time for work leading to more WFC (e.g., Wang, 2005; Gan, 2007). On the other hand, in terms of *stress from family*, previous studies found that the level of social support from family is negatively correlated with employees' WFC level (e.g., Chen et al., 2010; Zhao, 2015; French et al., 2018), and Chinese society has experienced a decline in social support from family (Tian, 2002; Xin et al., 2010). Specifically, the number of family member decreased by 1.30 from 4.41 in 1982 to 3.11 in 2016 (National Bureau of Statistics of China., 2017), which might lead to a reduced level of social support from family among Chinese employees. Moreover, the divorce rate in China has increased continuously (from 0.44 in 1985 to 3.02 in 2016), implying a more unstable and lonely family environment (Xin et al., 2010), which might make Chinese employees worry about their marital status. In addition, they also experienced increasing stress from supporting the elderly: the elderly dependency ratio in China has grown quickly and increased about 2-fold since 1980s (National Bureau of Statistics of China., 2017). These changes could lead to Chinese employees' sacrificing work time for family. Chinese employees also encountered increasing family economic stressors in recent decades: residents' consumption level has increased ~89-fold since 1980s (National Bureau of Statistics of China., 2017), which might cause employees to work hard for a living and cut the amount of time spending with their families. Therefore, for an interium summary in terms of those social indicators, the number of employees and college graduates could affect the intensity of employment competition, which in turn leads to changes in the employees' stress level from work. Meanwhile, the influence of divorce rate, family size, elderly dependency ratio and residents' consumption level accumulates to employees' stress level from family. Thus, we would hypothesize that changes in stress both from work and from family that are associated with the corresponding social indicators might be responsible for the increase WFC level for Chinese employees.

## Overview Of The Present Research

To test these hypotheses, the cross-temporal meta-analysis was performed to investigate the changes in WFC among Chinese employees. According to the proposition of dual-stress from work and family, certain social indicators might predict changes in the level of WFC over time. To examine such predictive effect, a time lag analysis was conducted to test the correlations between WFC and six social indicators related to dual-stress from work and family. Moreover, the findings of gender differences in WFC scores were controversial. Some studies (Frone et al., 1992; Behson, 2002; Zhou and Hao, 2009) showed that female employees have higher levels of WFC than males, while others found that the WFC among male employees was higher than among their female counterparts (e.g., Parasman and Simmers, 2001; Zhang, 2009). Therefore, we also performed the cross-temporal meta-analysis to investigate the changes in WFC for male and female employees, and conducted a general meta-analysis to explore whether there was gender difference in WFC.

## Methods

### Literature Search

#### Work-Family Conflict Scale

The Chinese version of the WFCS (Carlson et al., 2000) is the most popular scale to measure WFC. The WFCS consists of 18 items describing subjective feelings of WFC in two domains: (1) work interfering family (WIF, e.g., “*I can not carry out some ordinary family activities due to the influence of work*.”), reflecting that work can interfere with the ability to meet family demands; (2) family interfering work (FIW, e.g., “*Family life brings me anxiety and has an impact on my usual work performance*.”), reflecting that family can interfere with the ability to meet work demands (Bian et al., 2017; Ding et al., 2017; French et al., 2018). Responses are measured on a 5-point Likert Scale (1 = *strongly disagree*, 5 = *strongly agree*). Higher scores of WFCS indicate higher WFC.

#### Literature Search and Inclusion Rules

We selected the literature for the current study from the most influential academic literature databases in China: *CNKI, Wanfang*, and *Chongqing VIP*, which contain a large number of Chinese journal articles of all fields including natural sciences and social sciences as well as a considerable number of master's theses and doctoral dissertations since 1985. The search terms used to identify relevant studies were “employee”, “work-family conflict”, “psychological problems”, and “WFCS”. Further, using the same keywords, we searched the following three academic databases in English: *Web of Science, ProQuest Psychology Journals*, and *Elsevier*.

**Figure 1:**
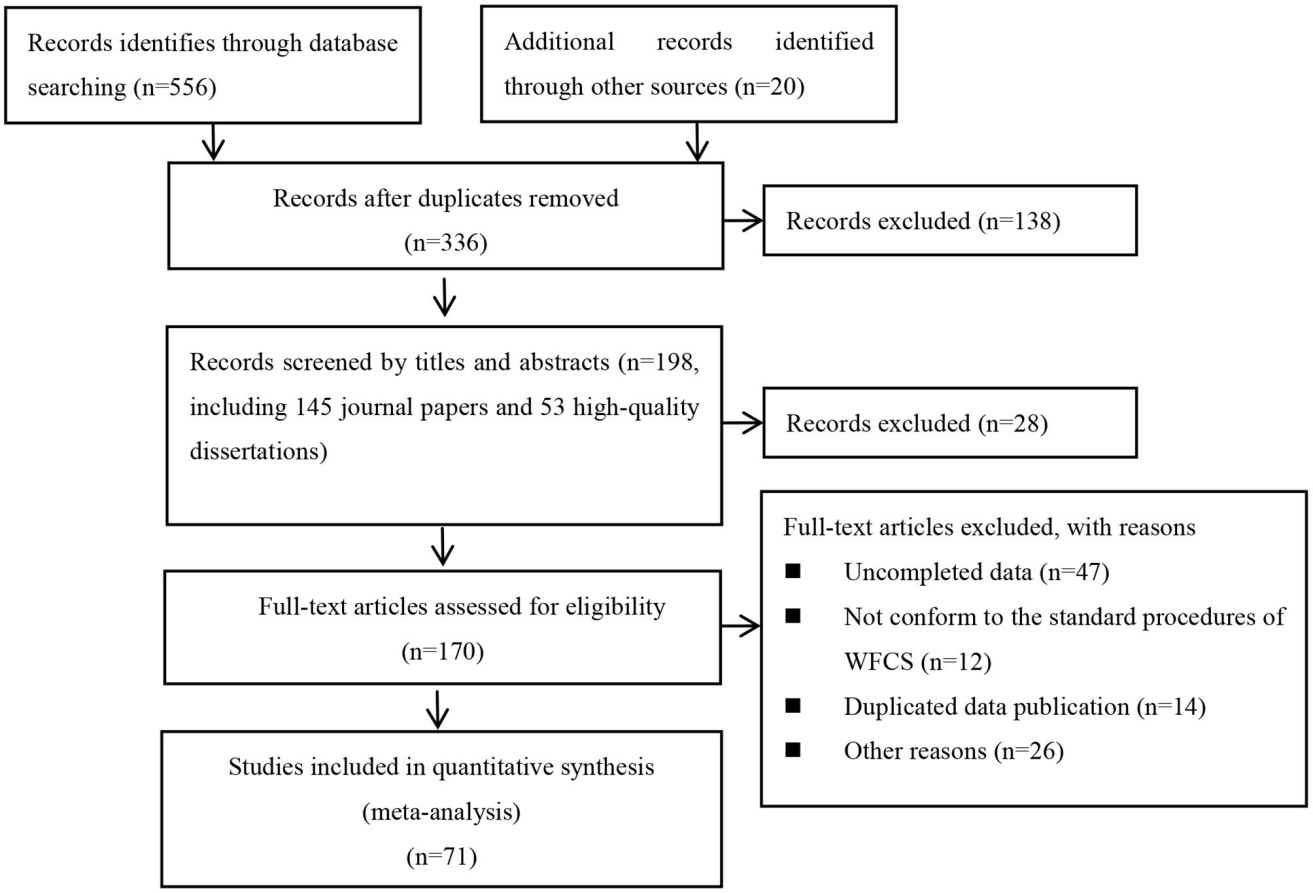
PRISMA flow chart for study selection.

Studies that could be included in the current cross-temporal meta-analysis must meet the criteria: (a) studies must have used all 18 items of the WFCS; (b) participants were employees involved in all walks of life (e.g., nurses, teachers, and bus drivers); (c) sample sizes and means for the total sample were reported; (d) participants should be from mainland China; (e) the sample size should be at least 30; (f) the study did not preselect participants who were clients at a counseling center. The PRISMA flowchart for study selection is shown in Figure 1.

#### Final Sample

This method yielded 71 studies of WFC, consisting of a total of 23,635 employees from 2005 to 2016 (the year of data collection). The minimum sample size is 116, and the maximum sample size is 1,273. There was no research of English language published conformance to the inclusion rules. Detailed information for included studies can be found in Table 1, and the references of all studies on meta-analysis are available in the references list.

**Table 1:** Description of work-family conflict studies included in the overall meta-analysis.

**Authors**	**Publication year**	***N***	**Region**	**Publication class**	**WFC**	**WIF**	**FIW**
Gan	2007	580	1	3	2.52 ± 0.59	NA	NA
Zeng et al.	2008	249	NA	2	2.43 ± NA	2.86 ± 0.84	2.00 ± 0.64
Cheng et al.	2008	1,273	NA	2	2.82 ± 0.50	3.29 ± 0.72	2.35 ± 0.59
Chen	2008	427	3	3	2.74 ± 0.64	2.97 ± 0.80	2.49 ± 0.74
Wang	2009	244	1	3	1.82 ± NA	1.80 ± NA	1.84 ± NA
Zhou and Hao	2009	543	2	1	3.04 ± NA	3.10 ± 0.65	2.98 ± 0.61
Zhang	2009	206	NA	3	3.15 ± NA	3.48 ± 0.87	2.81 ± 0.84
Chen et al.	2010	513	4	1	3.02 ± NA	3.49 ± 0.68	2.55 ± 0.71
Mu et al.	2010	305	1	2	2.69 ± 0.57	3.16 ± 0.78	2.22 ± 0.61
Fang	2010	142	NA	3	2.90 ± NA	3.20 ± NA	2.60 ± NA
Gao	2011	380	3	3	2.92 ± NA	3.29 ± 0.67	2.54 ± 0.52
Zhang and Liu	2011	387	1	2	2.85 ± NA	3.23 ± 0.74	2.47 ± 0.63
Zhou et al.	2011a	458	1	1	2.93 ± 0.62	3.56 ± 0.81	2.30 ± 0.66
Chen	2011	513	NA	3	3.02 ± NA	3.49 ± 0.69	2.55 ± 0.71
Zhou et al.	2011b	412	1	2	2.95 ± 0.62	3.46 ± 0.82	2.33 ± 0.66
Zhao	2011	578	NA	3	2.96 ± 0.58	3.63 ± 0.77	2.30 ± 0.68
Bi	2011	198	NA	3	3.15 ± NA	3.67 ± 0.55	2.63 ± 0.53
Zuo et al.	2011	260	NA	2	3.09 ± NA	3.53 ± NA	2.65 ± NA
Li et al.	2011	197	4	2	NA	2.64 ± NA	NA
Sun et al.	2011	207	NA	1	3.60 ± NA	NA	NA
Yan et al.	2011	426	3	1	2.98 ± 0.48	2.72 ± 0.26	3.24 ± 0.28
Zhang	2011	313	5	3	2.66 ± NA	2.76 ± 0.08	2.56 ± 0.37
Yan	2012	577	NA	3	3.16 ± 0.53	3.44 ± NA	2.89 ± NA
Shen et al.	2012	210	1	2	2.82 ± NA	2.98 ± 0.56	2.66 ± 0.50
Liu and Wu	2012	426	3	2	2.96 ± NA	3.34 ± 0.66	2.57 ± 0.54
Liu Y.	2012	376	4	3	2.93 ± 0.70	2.97 ± 0.78	2.95 ± 0.79
Sun	2012	727	NA	3	2.42 ± 0.81	NA	NA
Li	2012	116	NA	2	2.64 ± 0.84	NA	NA
He and Sun	2012	210	4	2	2.79 ± 0.50	3.13 ± 0.65	2.45 ± 0.64
Feng et al.	2012	273	4	1	2.97 ± NA	3.27 ± 1.01	2.66 ± 0.27
Li C. K. et al.	2012	197	4	3	NA	NA	NA
He	2012	209	2	3	2.72 ± 0.62	2.95 ± 0.70	2.49 ± 0.76
Li K. J. et al.	2012	574	3	1	2.89 ± 0.53	3.24 ± 0.67	2.54 ± 0.58
Liu X.	2012	170	5	1	2.63 ± 0.91	3.14 ± 0.99	2.80 ± 0.69
Pan and Chen	2012	250	3	2	3.11 ± NA	NA	NA
Zhang Z. F.	2013	256	NA	3	3.04 ± 0.49	3.18 ± NA	2.79 ± NA
Wang	2013	216	5	3	3.23 ± NA	NA	NA
Hu et al.	2013	514	4	1	3.01 ± 1.69	2.99 ± NA	3.03 ± NA
Wang et al.	2013	577	NA	2	3.04 ± 0.56	NA	NA
Zhang Y. X.	2013	300	1	3	3.20 ± NA	4.42 ± 0.33	1.98 ± 0.72
Zhao	2013	578	2	1	3.07 ± NA	3.83 ± 0.76	2.30 ± 0.68
Jiang	2013	138	NA	3	3.11 ± 0.65	3.10 ± NA	NA
Hong et al.	2013	230	NA	2	2.79 ± 0.50	2.91 ± NA	2.66 ± NA
Chen	2013	250	NA	3	3.11 ± 0.56	NA	NA
Liang	2014	244	NA	3	3.34 ± 1.09	NA	NA
Chen et al.	2014	234	5	2	3.23 ± NA	3.25 ± 0.86	3.21 ± 0.85
Huang	2014	119	1	3	3.31 ± 0.58	3.71 ± 0.69	2.91 ± 0.39
Rong et al.	2014	372	1	1	3.07 ± 0.68	3.35 ± 0.76	2.80 ± 0.72
Yang	2014	296	5	3	NA	2.63 ± 0.74	NA
Zhang and Qian	2014	139	1	3	3.09 ± NA	3.41 ± 0.72	2.77 ± 0.60
Zhu	2014	183	NA	3	2.53 ± NA	2.74 ± 0.88	2.32 ± 0.69
Xu	2015	432	3	3	3.57 ± 0.69	3.64 ± 0.71	3.50 ± 0.72
Xing	2015	276	3	3	NA	NA	NA
Wang	2015	476	NA	3	2.74 ± 0.67	3.00 ± 0.78	2.48 ± 0.77
Jia	2015	395	5	3	2.57 ± NA	2.58 ± 0.61	NA
Qi	2015	467	NA	2	3.19 ± NA	3.16 ± 0.79	3.21 ± 0.77
Cao et al.	2015	482	NA	2	3.05 ± 0.51	3.69 ± NA	2.40 ± NA
Xie et al.	2015	261	NA	1	NA	NA	NA
Zhao	2015	205	1	3	NA	NA	2.66 ± 1.04
Yuan	2016	237	NA	3	2.66 ± NA	NA	NA
Ren	2016	296	NA	3	NA	3.80 ± 1.21	NA
Song and Guo	2016	207	4	3	2.93 ± 0.29	2.94 ± NA	2.93 ± NA
Yang	2016	286	3	3	2.70 ± NA	2.72 ± NA	2.67 ± NA
Bian et al.	2017	133	2	2	2.91 ± 0.59	2.99 ± 0.79	2.84 ± 0.76
Ding et al.	2017	159	3	2	2.74 ± 0.60	3.25 ± 0.78	2.22 ± 0.57
Hao et al.	2017	200	2	2	3.15 ± 0.48	3.55 ± 0.61	2.75 ± 0.27
Ren et al.	2017	306	3	1	3.17 ± 0.54	3.62 ± 0.62	2.72 ± 0.70
Zhang and Sun	2017	344	5	2	4.08 ± 0.68	4.16 ± 0.65	3.99 ± 0.53
Cheng	2017	277	5	3	3.56 ± 0.78	NA	NA
Yang	2017	209	5	3	3.21 ± 0.69	3.33 ± 0.73	2.97 ± 0.65
Tang	2017	215	4	2	2.99 ± 0.67	3.39 ± 0.84	2.60 ± 0.73

*N, sample size; WFC, work-family conflict total score; WIF, work interfering family score; FIW, family interfering work score; region, Region: 1 = east; 2 = northeast; 3 = middle; 4 = west; 5 = mixed; publication class, 1= core journal; 2 = publication from other academic sources; 3 = dissertations and master's theses; NA, missing values*.

### Main and Control Variables Coding

The average score and standard deviation (*SD*) of WFC, sample size, and the year of data collection were recorded in each study. The year of data collection was coded as 2 years before publication if not specified in the article (Twenge, 2000; Xin et al., 2010). If an article reported sample mean and *SD* for different subgroups (i.e., means by gender), the overall mean and *SD* were calculated weighted by sample size of each subgroup. Furthermore, publication class, region, and gender ratio in each study were recorded and used as controls in the analysis, because they may confound with birth cohort. Consistent with previous studies (e.g., Xin and Zhang, 2009; Xin and Xin, 2016, 2017), region was coded into *East, Northeast, Middle, West* (in descending order of economic level), or *Mixed* (i.e., participants in one study were selected from more than 1 region). Publication class was coded into first (journals from CSSCI and CSCI), second (other journals), and third class (master's theses and doctoral dissertations). Publication class was controlled for to avoid the influence of publication bias. Namely, studies with statically significant results would have higher chance to be accepted for publication than those with non-significant findings (Xin et al., 2010; Xin and Xin, 2016, 2017).

### Sources for Social Indicators

Consistent with previous research (e.g., Xin and Zhang, 2009; Xin and Xin, 2016), we chose the number of employees and college graduates as two indicators of the level of *stress from work* and selected family size, divorce rate, elderly dependency ratio, and residents' consumption level as four indicators of the level of *stress from family*. In the present study, these two types of important social indicators that might reflect macro social changes in Chinese society and account for changes in employees' WFC. All of these indicators were obtained from *China Statistical Yearbook* (CSY; National Bureau of Statistics of China., 2017).

### Data Analysis Strategy

Unlike traditional meta-analysis, the cross-temporal meta-analysis does not focus on an effect size for each study and does not need use the comprehensive meta-analysis (CMA). Instead, the main focus of the cross-temporal meta-analysis is to investigate the change in mean scores on psychological measures across time. Therefore, to examine the changes in WFCS scores over time, correlations were conducted between mean WFCS scores of each study and year of data collection, weighted by sample size of each study (e.g., Twenge and Campbell, 2001; Twenge and Foster, 2010; Xin and Xin, 2016, 2017). We performed the analyses using SPSS, and reported standardized βs. For a study that only reported a sample mean without *SD*, we included this study to examine the mean-level WFCS changes to make best use of data, but only included those with both means and *SD*s to estimate the magnitude of the changes of WFC scores.

Then, we used three weighted regression equation and the average standard deviation (*SD*) of the individual samples to calculate the magnitude of the changes of work-family conflict scores (WFC, WIF, FIW). The following regression equation was used to calculate the mean scores for a specific year: *y* = *b x* + *c*, where *b* = the unstandardized regression coefficient, *c* = the intercept or constant, *x* = the year of data collection, and *y* = the predicted mean score (WFC, WIF, FIW). These equations yielded the expected average WFC, WIF and FIW scores for particular years. We computed the average *SD* by averaging the within-sample *SD*s reported in the studies. It is important to note that this method is likely to avoid the ecological fallacy, also known as ecological correlations or alerting correlations (Rosenthal et al., 2000), which occurs when the magnitude of change is calculated using the variation in mean scores rather than the variation within a population of individuals. This exaggerates the magnitude because mean scores do not differ as much as individual scores. The method used here, in contrast, uses the *SD* of the individual studies to capture the variance of the scale among a population of individuals.

Finally, we performed a time lag analysis based on previous research (e.g., Xin and Xin, 2016) to determine whether two types of social indicators could explain changes in WFC. Two indicators of the level of *stress from work* (number of employees and college graduates) and four indicators of the level of *stress from family* (family size, divorce rate, elderly dependency ratio, and residents' consumption level) of each year was matched with the WFCS mean of each study of the year in two ways: the year of data collection, and 5 years before the data were collected. For instance, a data point for WFCS score of each study from 2010 was matched with each social indicator from 2005, 2010. Three regression analyses of each social indicator were conducted by weighting sample size. We reported the standardized βs, representing the correlations between social indicators related to *dual-stress from work and family* and WFC, weighted by sample size. According to Twenge (2000), if social indicators of *dual-stress from work and family* can explain the changes in WFC over time, the correlations should be significant when WFCS scores are matched with social indicators lagged several years ago. Furthermore, if WFC and social indicators have concurrent associations, there should be significant correlations between the two variables at the year of data collection.

## Results

### Correlations Between Mean Scores of Work-Family Conflict and Year

**Figure 2:**
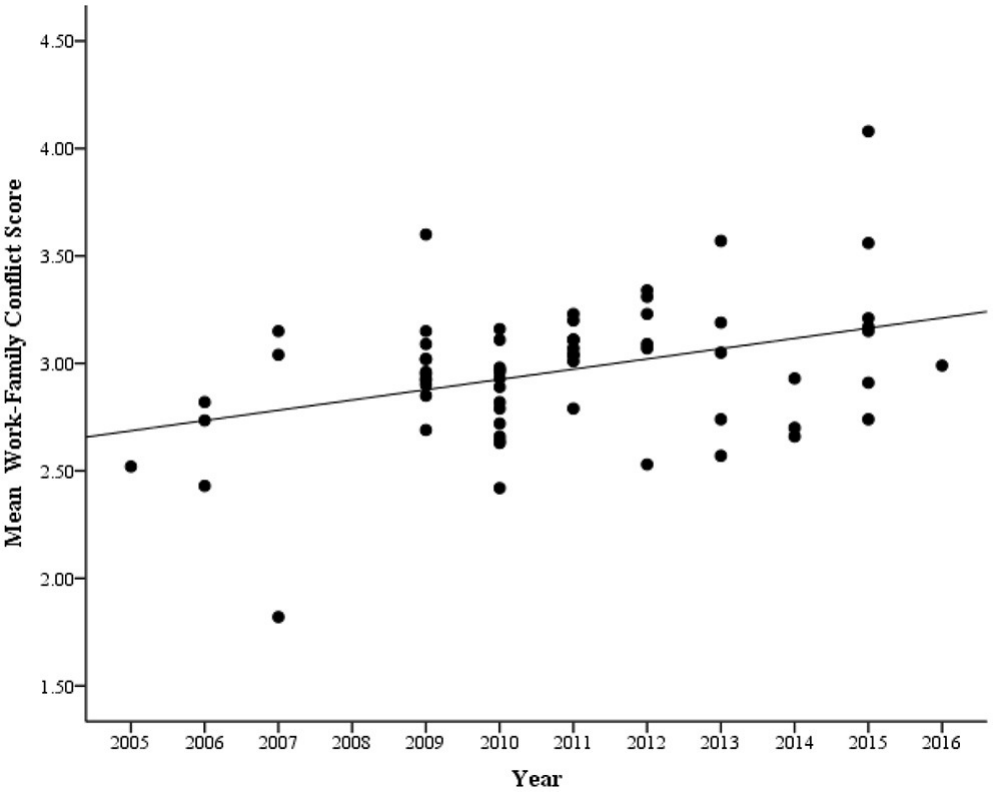
Changes in work-family conflict of Chinese employees based on cross-temporal meta-analysis, 2005–2016.

**Figure 3:**
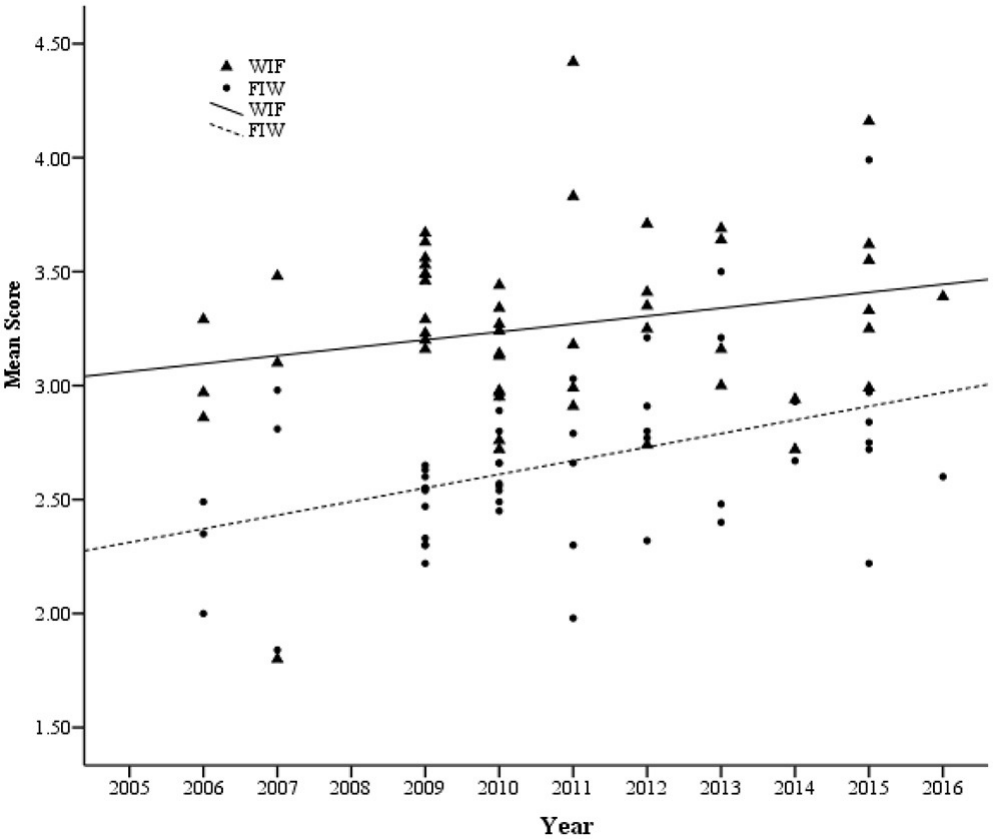
Changes in WIF and FIW scores of Chinese employees based on cross-temporal meta-analysis, 2005–2016. WIF, work interfering family; FIW, family interfering work.

Figures 2, 3 indicated that Chinese employees' scores on WFCS (WFC, WIF, FIW) increased gradually from 2005 to 2016. Meanwhile, we conducted a Steiger's *Z* test to investigate whether the trends of FIW and WIF changes were statistically different, and found that distinct patterns of change for FIW and WIF was not observed (*Z* = −1.17, *p* > 0.05). Furthermore, in order to demonstrate how WFC scores of Chinese employees have changed from 2005 to 2016, we conducted three regressions with WFC, WIF, FIW scores (as dependent variables), and year of data collection (as independent variables) with all controlling variables (gender ratio, publication class, and region) as covariates. As shown in Table 2, both WFC and FIW scores were significantly positively correlated with the year of data collection. However, no significant correlation was found between WIF score and the year of data collection. That is, there was a significant increase with Chinese employees' WFC and FIW scores from 2005 to 2016, but not with their WIF scores. The correlations were also very similar when gender ratio, publication class and region were controlled and sample size was weighted (see Table 2). Thus, Chinese employees' WFC score (especially FIW) showed an evident increase over the past 12 years.

**Table 2:** Correlations between the year of data collection and employees' work-family conflict scores, weighted by sample size, 2005–2016.

**Variables**	**Bivariate**		**Weighted**			
	***r***	**95% CI**	**β**	***SE***	**95% CI**	***R*^**2**^**
WFC	0.38^**^	[0.15, 0.57]	0.42^**^	0.01	[0.20, 0.61]	0.18
WIF	0.21	[−0.06, 0.45]	0.17	0.02	[−0.10, 0.42]	0.03
FIW	0.41^**^	[0.16, 0.62]	0.44^**^	0.02	[0.19, 0.64]	0.19

*WFC, work-family conflict total score; WIF, work interfering family score; FIW, family interfering work score; CI, confidence interval*.

** *p <0.01*.

Single-gender means were also analyzed separately when they were reported, with controls included. Female employees' WFC score was not significantly correlated with year (β = 0.32, *SE* = 0.02, *p* = 0.09, 95% CI = [−0.03, 0.60], *R*^2^ = 0.11), whereas male employees' WFC score was significantly correlated with year (β = 0.48, *SE* = 0.03, *p* = 0.03, 95% CI = [0.09, 0.75], *R*^2^ = 0.23). The positive correlations between FIW score and year among male and female employees were both significant (β_men_ = 0.57, *SE* = 0.03, *p* = 0.03, 95% CI = [0.08, 0.84], *R*^2^ = 0.32; β_women_ = 0.48, *SE* = 0.02, *p* = 0.03, 95% CI = [0.07, 0.75], *R*^2^ = 0.23). In addition, for male employees, the correlation between WIF score and year was not significant (β = 0.27, *SE* = 0.03, *p* = 0.30, 95% CI = [−0.23, 0.66], *R*^2^ = 0.07); and the correlation for female employees was also not significant (β = 0.06, *SE* = 0.02, *p* = 0.77, 95% CI = [−0.33, 0.43], *R*^2^ = 0.01). In short, WFC score on the WFCS increased across birth cohort among male and female employees, with a slightly stronger relationship for male employees than female employees.

To examine gender difference in WFC, in total 21 studies that reported means by gender were analyzed by a general meta-analysis using SPSS (gender difference was indicated by the effect size *d* for this analysis). The formulae are written as follows: $\bar{\text{d}}$ = Σ*W*_*i*_*d*_*i*_/Σ*W*_*i*_, *W*_*i*_ = 2*N*_*i*_/(8+$d_{i}^{2}$), *d* = (*M*_female_−*M*_male_)/*SD*, $SD=\sqrt{\left[\left(n_{e}-1\right)S_{e}^{2}+\left(n_{c}-1\right)S_{c}^{2}\right]/\left(n_{e}+n_{c}-2\right)}$, where *W*_i_ is the weight of all studies, *N*_i_ is the total sample size, *d* is the effect size of a single study, and *SD* is the combined standard deviation of male and female employees, n_e_/n_c_ and S_e_/S_c_ are the sample size and the standard deviation of male or female employees, respectively. Results showed that mean effect sizes of gender difference in WFCS scores (*d*_WFC_ = −0.05, *d*_FIW_ = −0.15, *d*_WIF_ = −0.02, respectively) were small according to Cohen's (1992) standards (i.e., effect size is large when *d* = 0.80, medium when *d* = 0.50, and small when *d* = 0.20), which suggested that there was no significant difference in WFC between males and females.

The above results showed that WFC of Chinese employees increased gradually. However, exactly how much has WFC increased? To further examine the amount of changes in WFC scores, three weighted regression equations were performed to predict the mean scores of the first and the last year of included studies. During 2005–2016, the mean scores of WFC, FIW, and WIF increased by 0.50, 0.65, and 0.27, respectively. The average *SD*s of WFC, FIW, and WIF reported for the individual samples were 0.65, 0.64, and 0.72, respectively. Thus, WFC, FIW, and WIF scores increased 0.77, 1.02, and 0.38 standard deviations from 2005 to 2016, that is *d*_WFC_ = 0.77, *d*_FIW_ = 1.02, and *d*_WIF_ = 0.38 (*d* = (*M*_2016_−*M*_2005_)/*M*_SD_, where *M*_2016_ and *M*_2005_ represent the mean score in the last and first year, respectively, and *M*_SD_ represents the averaged *SD*). Therefore, according to Cohen's (1992) guidelines, *d*_FIW_ = 1.02 should be considered a large effect size, *d*_WFC_ = 0.77 should be considered a medium effect size, and *d*_WIF_ = 0.38 should be considered a small effect size. If we converted the *SD* into percentile scores, the result is also informative. If the average Chinese employees in 2005 scored at the 50^th^ percentile of the distribution, the average employees' WFC, FIW, and WIF in 2016 were in the 78^th^, 85^th^, and 65^th^ percentile (assuming a normal curve), respectively. The *d* value was then converted (0.77, 1.02, and 0.38) into variance explained by year and found that the proportion (*r*^2^ = *d*^2^/(4+*d*^2^)) was ~12.91, 20.64, and 3.48%. That is, birth cohort explains 12.91, 20.64, and 3.48% of the variance in WFC, FIW, and WIF, respectively.

### Correlations of Employees' Work-Family Conflict With Social Indicators Over Time

The direct correlations between WFC scores and social indicators provided possible explanations of the increase in Chinese employees' WFC. As mentioned above, two types of social indicators related to *dual-stress from work and family* were matched with WFC data in two ways: 5 years prior to data collection, and the year of data collection (see Table 3). If the correlations were significant between social indicator scores from 5 years prior or the year of data collection and WFC scores, social indicators could predict WFC changes. It is believed that the time lag of 5 years helped us to determine the predictive effect of social indicators on WFC scores.

**Table 3:** Correlations between social indicators and employees' work-family conflict scores, weighted by sample size, 2005–2016.

**Social indicators**	**WFC**		**WIF**		**FIW**	
	**β**	**95% CI**	**β**	**95% CI**	**β**	**95% CI**
**FIVE YEARS PRIOR**						
Family size	−0.26*	[−0.48, −0.02]	−0.04	[−0.30, 0.23]	−0.45^**^	[−0.65, −0.20]
Divorce rate	0.42^**^	[0.20, 0.61]	0.20	[−0.07, 0.44]	0.42^**^	[0.17, 0.62]
Residents' consumption level	0.40^**^	[0.17, 0.59]	0.16	[−0.11, 0.41]	0.43^**^	[0.18, 0.63]
Elderly dependency ratio	0.45^***^	[0.23, 0.63]	0.21	[−0.06, 0.45]	0.41^**^	[0.16, 0.62]
Number of employees	0.45^***^	[0.23, 0.63]	0.19	[−0.08, 0.43]	0.45^**^	[0.20, 0.65]
Number of college graduates	0.41^**^	[0.19, 0.60]	0.15	[−0.12, 0.40]	0.44^**^	[0.19, 0.64]
**ACTUAL YEAR**						
Family size	−0.19	[−0.42, 0.06]	−0.02	[−0.28, 0.25]	−0.32*	[−0.55, −0.05]
Divorce rate	0.40^**^	[0.17, 0.59]	0.16	[−0.11, 0.41]	0.44^**^	[0.19, 0.64]
Residents' consumption level	0.42^**^	[0.20, 0.61]	0.16	[−0.11, 0.41]	0.44^**^	[0.19, 0.64]
Elderly dependency ratio	0.41^**^	[0.19, 0.60]	0.17	[−0.10, 0.42]	0.43^**^	[0.18, 0.63]
Number of employees	0.42^**^	[0.20, 0.61]	0.16	[−0.11, 0.41]	0.44^**^	[0.19, 0.64]
Number of college graduates	0.42^**^	[0.20, 0.61]	0.17	[−0.10, 0.42]	0.42^**^	[0.17, 0.62]

*WFC, work-family conflict total score; WIF, work interfering family score; FIW, family interfering work score; CI, confidence interval*.

* p < 0.05

** *p < 0.01*,

*** *p < 0.001*.

As shown in Table 3, correlations were analyzed between social indicators (5 years prior, and the year of data collection) and Chinese employees' WFC. Results demonstrated that the most significant correlations appeared between WFC and FIW scores and six social indicators from 5 years prior. Correlations between WFC and FIW scores and 6 social indicators from the year of data collection were also significant. In addition, the correlations between FIW scores and six social indicators from 5 years before or the year of data collection were not significant (as shown in Table 3). That is, six social indicator scores from 5 years before or the year of data collection predicted the change in WFC (especially FIW), which indicated that the rise in the number of employees, number of college graduates, divorce rate, elderly dependency ratio, residents' consumption level, and decline in family size may be some crucial predictors of the increase of WFC in China.

## Discussion

### Changes in Work-Family Conflict in the Past 12 Years

We performed a cross-temporal meta-analysis of 71 studies to investigate changes in levels of WFC of Chinese employees during 2005–2016. An overall increase was found in scores on WFCS of Chinese employees over time. Both the scores for WFC and FIW increased significantly over time. However, the increase in WIF was not significant. These results supported our hypothesis that WFC has increased over the past 12 years, suggesting that the macro socio-cultural environment may have substantial effects on individuals' psychological characteristics.

As mentioned above, the increase in the number of the employed population and college graduates in China might boost employees' stress level from employment competition and unemployment risk. To avoid being laid off, employees must engage more in work to improve their work competence, which inevitably requires long working hours and ineluctably cause different degrees of work engagement. It was found that people with high work engagement have high WFC (e.g., Greenhaus and Kopelman, 1981) because high engagement with work involves less time spent with their family, hindering them from fulfilling their family responsibilities.

In addition, the increase in WFC may be related to the decrease in social support, which is in line with previous studies showing that WFC was negatively correlated with social support (e.g., Ford et al., 2007). Moreover, some researchers found that the reduction in social support for work increased levels of WIF and FIW (French et al., 2018). In recent decades, the largely declined social connectedness in China and the collapse of traditional Chinese family system might explain the reduction both in the perceived level of social support for Chinese employees and in the quality of their contact with family members and other people (Xin et al., 2010; Xin and Xin, 2016). Consequently, higher WFC occurred. Moreover, results of the current research indicated that both the growth rate and the variation explained by year in FIW were higher than those in WIF. This difference may be due to the belief that family is more important than work (Wang, 2009). However, people generally tend to sacrifice family time for work, which intensifies the WFC and reduces support from family members. Meanwhile, the one-child policy in China limited population growth until 2015, and the population aging problem in China is becoming worse, which might lead to faster growth in the level of FIW than in the level of WIF.

It is worth noting that the changes in female employees' WFC scores were not significant, which might be consistent with the decline in the desired fertility of the Chinese population (Hou et al., 2014). Female employees' stressors come mainly from bearing and taking care of children; however, their desired fertility has decreased significantly, which might alleviate WFC among female employees. Moreover, the increasing trends in WFC scores for FIW were stronger among male employees than female employees, which could be due to the traditional cultural view that men are supposed to be more independent. Women instead, should be provided with more social support than men (Chen and Zhang, 2012; Xin and Xin, 2016). Gender differences in the means of each type of WFC were small indicated by effect size, which may be because they gradually have equal status both in family and education (Chen, 2008), and the one-child policy has reduced the number of children for whom women need to care.

### The Predictive Effect of Social Indicators on Work-Family Conflict

Previous studies have mostly involved the *proximal* factors (e.g., gender and personality, etc.) influencing WFC of each subject at the individual level, while ignoring the *distal* factors (e.g., the number of employees and residents' consumption level, etc.) influencing WFC of a group at the macro level. Therefore, the focus of the present study has been on the *distal* factors at the macro level influencing WFC. This study provided a special opportunity to investigate the predictive effect of two types of social indicators (*distal* factors at the macro level) on WFC over time. Although prior research has shown that some social indicators (i.e., social support, organizational support) were correlated with WFC among employees (e.g., Zhang, 2009; Chen et al., 2010), they only used cross-sectional design to investigate the intrapersonal associations among these variables. There was no study examined the relationship between WFC and social indicators over time at the group level.

Based on the proposition of *dual-stress from work and family*, six social indicators of both stress from work and family were considered as facts of the socio-cultural environment, which may be potential predictors of changes in WFC. Therefore, we investigated the predictive effect of six social indicators on Chinese employees' WFC over time by adopting the time lag analysis to compute the correlations of WFC and two types of social indicators related to stress both from work (the number of employees and college graduates) and from family (family size, divorce rate, elderly dependency ratio, and residents' consumption level). As shown in Table 3, two types of social indicators of 5 years before and the year of data collection were significantly correlated with WFC and FIW scores, whereas, the correlations between WIF and social indicators were not significant. Possible explanation for these results is that changes in the stress from work and family were associated with WFC (especially FIW). Specifically, on the one hand, regarding the stress from work, the rise in the number of employees and college graduates might increase pressure from employment competition, which could lead to the rise in employees' WFC. On the other hand, in terms of the stress from family, the decline in social support (i.e., family size), the increased pressure from spousal relationships (divorce rate), supporting the elderly (elderly dependency ratio) and family economic situation (residents' consumption level) might produce higher levels of WFC (especially FIW) among Chinese employees. In a word, the macro external stress brought by the rapid growth of China's economy led to the rise of FIW perceived by employees, but not for WIF.

### Implications and Limitations

Our findings from the present research could have important theoretical and practical implications. Most importantly, this research provided evidence to confirm the predication that WFC of Chinese employees has increased in the past 12 years using the cross-temporal meta-analysis. The current study found that the increasing level of WFC among Chinese employees was associated with six social indicators related to stress both from work (i.e., number of employees, number of college graduates) and from family (i.e., family size, divorce rate, elderly dependency ratio and residents' consumption level) over time. These findings might not only contribute to the knowledge on the *distal* factors at the macro level influencing WFC, but also suggest that it is necessary to make stress-reducing policies to reduce WFC in China. Therefore, to reduce the WFC level of employees, researchers should investigate the factors influencing WFC of employees and provide some theoretical and empirical evidences to reduce the WFC level of employees; clinicians should focus on the outcome variables of WFC (especially psychological problems) and offer more effective mental health services (such as lectures on coping with WFC) and design interventions and therapeutic techniques to relieve pressure and reduce mental health problems; policymakers should pay attention to the mental health of employees and adopt effective ways such as Employee Assistance Programs (EAP) to reduce the level of perceived WFC of employees.

Despite these implications, several limitations should be acknowledged. Although the most commonly used assessment of WFC in employees in China is the WFCS, other instruments (e.g., Greenhaus and Beutell, 1985; Frone et al., 1992) have also been employed to examine WFC in this population. Therefore, we suggest that future studies analyze results yielded by different scales in order to deepen the understanding of WFC. In terms of methodology, the effectiveness of the cross-temporal meta-analysis method in reducing ecological fallacy was challenged by some researchers (e.g., Trzesniewski et al., 2008). Thus, we suggest that future research should verify the reliability of the results of the current study using the method proposed by Trzesniewski et al. (2008). Moreover, the present research limited its conclusions to Chinese employees. Future research should investigate whether WFC has also changed in other countries with rapid economic development. In addition, because the employees in the present sample were of different ages and our analysis covered a period of 12 years, it was difficult to distinguish cohort effects from those of age and time period. Unfortunately, few of the original publications employed by the present study provided information regarding the details of age or the WFC scores across different age groups. As this is a limitation, future research should examine this issue by improving current methods or developing new ones.

## Data Availability Statement

The datasets generated for this study are available on request to the corresponding author.

## Author Contributions

SX wrote this manuscript. YZ collected data. ZX revised this manuscript.

### Conflict of Interest

The authors declare that the research was conducted in the absence of any commercial or financial relationships that could be construed as a potential conflict of interest.
